# Incorporating Generative AI Into a Health Informatics Curriculum to Build 21st Century Competencies: Multisite Pre-Post Study

**DOI:** 10.2196/76507

**Published:** 2025-12-16

**Authors:** Freddie Seba, Miriam Isola, Laura Mills, Mohan Zalake, Jacob Krive

**Affiliations:** 1 Masters of Science in Digital Health Informatics School of Nursing and Health Professions, University of San Francisco, 2130 Fulton Street, San Francisco, CA, 94117, United States, 1 415-422-5555; 2 School of Education, Leadership Studies Program, University of San Francisco, 2350 Turk Blvd.San Francisco, CA, 94118, United States, 1 (415) 422-2204; 3 Department of Biomedical and Health Information Sciences, University of Illinois Chicago, Chicago, IL, United States

**Keywords:** generative artificial intelligence, student engagement, capstone experience, generative AI ethics, pedagogical innovation, generative AI, artificial intelligence, AI

## Abstract

**Background:**

We designed learning assignments for students to develop knowledge, skills, and professional attitudes about generative artificial intelligence (AI) in 2 different Master’s level courses in health informatics. Our innovative approach assumed that the students had no technical background or experience in using generative AI tools.

**Objective:**

This study aims to offer generalizable methods and experiences on integration and assessment of generative AI content into the higher education’s health informatics curricula. The study’s central driver is the preparation of graduate students with generative AI tools, skills, ethical discernment, and critical thinking capacities aligned with the rapidly shifting job-market requirements, independent of graduate students’ backgrounds and technical expertise.

**Methods:**

During the semester, students completed a pretest and posttest to assess knowledge about generative AI. Reflections explored their expectations and experiences using generative AI to complete their assignments and projects during the semester. Strong emphasis was placed on building skills and professional attitudes by using generative AI. Student engagement in behavioral, emotional, and cognitive domains was explored via detailed analysis of student reflections by faculty.

**Results:**

Students at the University of Illinois Chicago increased their knowledge about generative AI from 81% to 93% through research of the basic generative AI concepts, as evidenced from outcomes of the open-book pre-and posttests given at the beginning and end of the capstone course. University of San Francisco students also improved from 77% to 80% by the end of the semester. Faculty analysis of student reflections upon completion of the course revealed primary interests in the essentials of generative AI, AI transformations to information and knowledge, and organizational changes influenced by AI adoption in the health care organizations, with ethics being a primary driver of students’ interests and engagement.

**Conclusions:**

Data from student reflections provided insight into generative AI skills that students developed and that health informatics programs can consider incorporating into their curricula. Building competencies in generative AI will prepare students for the 21st century workforce and enable them to build skills employers are seeking in the new digital health environment.

## Introduction

The current decade has seen much technological progress, but one of the most impactful and controversial areas has been generative artificial intelligence (AI). Since AI was first discussed in the 1950s [1] and especially since it exploded onto the technological scene in the 2020s, it has been a source of both wonder and fear about how it might help, hinder, and otherwise permanently alter human life. Today, in 2025, AI is a household term and a daily topic of news and discussion. It is easy to imagine a future in which AI is even more ubiquitous than it already is, but two of the most talked-about areas of AI incorporation today are education and health care.

AI use has both supporters and detractors in education and health care. Proponents of AI in education tout its potential to individualize learning experiences and thereby increase student engagement; they also advocate for AI’s many supportive functions such as analyzing and managing various student data and streamlining administrative tasks [2]. Meanwhile, AI has been used in health care since the development of a glaucoma consultation program at Rutgers University in 1976 [1]. Since then, exponential advancements in AI system design have led to a proliferation of clinical uses. ChatGPT, a popular tool developed by Open AI, for example, is being used in applications such as answering patient questions; assisting in clinical data analysis and decision making; responding to emergencies; assisting in practice management; and assisting in data management and other aspects of medical research [3].

Along with the advantages and benefits of generative AI’s educational and health care applications, each presents specific challenges and raises questions. Detractors of generative AI in education cite the potential for students to cheat with AI. Some also warn against students leaning too heavily on AI to the diminishment of their own critical thinking abilities [2]. In both education and health care, questions arise about output accuracy plus such ethical issues as data privacy and output bias, among others.

Health informatics lies at the intersection of technology and health care, and as health informatics educators, we realize that whether we welcome it or fear it, AI will play a large role in our students’ futures. In August of 2024, 28% of the US population aged 18‐64 years reported using AI at work, with 1 in 9 using it on a daily basis [4]. More precisely, our students will graduate into a health care profession that is already regularly using AI, and they will work for employers who expect that they are well prepared to use it in their jobs. In fact, employers already prioritize AI competencies over prior non-AI experience [5] in the job candidates’ profiles and believe AI skill sets are more important than many others [6]. Employers want graduates who can design, implement, and then manage needs-based AI systems; they also want graduates equipped with the specialized knowledge and skills to monitor and maintain those systems [7]. Job seekers need proficiency in using AI for data cleaning and management, performance management, and predictive analytics, among other knowledge and skills, to compete for positions in the most AI-advanced companies [8] Knowing that technology, including AI, advances vastly more quickly than official university curricula, we felt it imperative to transition some of our assignments so our students could begin using AI in a health informatics context now.

Let us first define what we mean by AI literacy. AI literacy is the critical ability to understand and mindfully interact with AI. It is more than just understanding how AI applications operate at a technical level. It is about understanding their functional level, benefits, challenges, and limitations [8]. AI literacy encompasses understanding the technical aspects of how it operates but also involves using critical thinking to assess its impact on humans, systems, society, and the environment [9]. AI literacy extends beyond a conceptual understanding of the theory behind it to also encompass the ability to ethically and responsibly use it to address problems, conduct research, or enhance productivity and growth. It is about actively using AI tools to solve problems and, in doing so, feeling engaged and interested in the process [10]. According to recent research, developing AI literacy involves 3 integrated skills: understanding fundamental AI concepts, evaluating the broader societal impacts of AI, and effectively using AI technologies in everyday contexts [9*].*

We represent two very distinct Master’s in Health Informatics programs. University of Illinois Chicago (UIC) is an all-online, asynchronous program founded in 1999 and has been completely online since 2010. Many of our students are returning to school after time spent working; thus, they are older than traditional graduate students, and many already possess significant experience in health care, business, or technical fields. The UIC program has an overall focus on population-level social informatics and offers students courses and specialization options in data science, mHealth, and leadership.

University of San Francisco (USF) is a hybrid program. Students spend over half their time in-person on campus and the remainder of their time online. The USF’s Master of Science in Digital Health Informatics is a young, dynamic program designed for small cohorts of approximately ≤20 students, offering personalized education through a combination of hybrid and in-person formats. Many students are recent bachelor’s degree recipients with backgrounds in public health, business, health professions, nursing, medicine, and technology. The USF program focuses on data analytics, digital health, and clinical informatics, as well as offering course options in public health informatics, clinical leadership, and nursing informatics. The Master of Science in Digital Health Informatics program equips students with essential skills in health care, data science, and technology, emphasizing practical and project-based learning. Graduates typically pursue careers in hospitals, digital health startups, health care analytics companies, pharmaceutical companies, nonprofit organizations, and clinical research institutions, leveraging their specialized training to improve health care outcomes and informatics solutions.

In our endeavor to rapidly infuse AI into our curricula, we focused on 4 knowledge domains of AI competencies for health informatics education: essentials of AI; applications of AI to health informatics; AI transformations to information and knowledge; and organizational change and adoption of AI within the health care organization. We based these categories on prior work done in the development of a health data science concentration within a health informatics curriculum [11]. As part of that work, a list of 50 skills and competencies, categorized into 4 knowledge domains, was developed based on input from health informatics professionals and industry experts, health informatics faculty, and analysis of the health informatics labor market. We modeled the 4 knowledge domains of AI competencies for health informatics education on the original 4 knowledge domains for health data science. Within each of these AI competency categories, we listed knowledge, skills, and attitudes we believed our programs would need to provide for our students, leaving room for future ideas. Our new work builds on the previous work, incorporating generative AI skills and competencies into the same knowledge domains. The domains, skills, and competencies then enabled us to develop generative AI assignments.

During the fall 2024 semester, each program incorporated a generative AI assignment into a course, assessing each student’s preliminary knowledge of AI, their knowledge of AI at the conclusion of the assignment, and then each student’s reflections on AI. UIC students participated in this study during BHIS 593, their capstone experience, the culminating course in the Master’s in Health Informatics. This course requires students to research a topic of interest over the 16-week semester, delivering a paper or project at the end. The goal is for students to synthesize their learnings from their degree program and demonstrate competency as health informaticians.

USF students participated in this study during HS 633, *Exploring Generative AI Ethics: Intersection of Education and Health Ecosystems,* which they took approximately half-way through their master’s program. This course, a hands-on workshop, incorporates the latest AI literature and tools and focuses on generative AI, AI ethics and governance concepts, AI applications, use cases, frameworks, and AI policies with special emphasis on health care. The goal is to equip students with foundational knowledge and practical skills in generative AI, preparing them to navigate the Silicon Valley tech ecosystem and the traditional health care landscape [11]. USF’s generative AI Ethics course (HS 633) was conceived not as a technical deep dive into generative AI models and methods but as a framework-building course that foregrounds ethics and governance. This course was offered for the first time in Fall 2024 to coincide with this research study.

Our research questions for this multisite study were:

1. Did students learn (develop knowledge) about generative AI by doing the assignments?
2. Did students say they developed generative AI skills and professional attitudes by completing the assignments?

## Methods

### Design, Setting, and Participant Recruitment

This was a multisite study of assessment of assignments completed by Master of Health Informatics students at UIC and USF campuses in the fall of 2024. Across both sites, a total of 17 students participated in the research.

### Ethical Considerations

The institutional review boards (IRB) of the UIC and USF approved this study under the exempt research determination under the UIC ID STUDY2024-0927 and USF’s ID 2167. This was a multisite study of the assessment of assignments completed by Master of Health Informatics students at UIC and USF campuses in the fall of 2024. Across both sites, a total of 17 students participated in the research.

### UIC Program

At UIC, the research was implemented in the online health informatics curricula for Biomedical Health Information Sciences in the capstone course required to complete the Master of Science in Health Informatics (MSHI) program. There were 10 (59%) participants from UIC. Students chose from the 4 areas of generative AI practice and developed their specific topics working with faculty to define the scope and deliverables for their project. Topics explored real-world questions of interest for which students developed a blueprint or prototype solution or a use case. Table 1 contains information about the 4 topic areas.

**Table 1.** Generative artificial intelligence (AI) topics.

Topics	Focusing questions	Description
Clinical uses	How is generative AI being used to augment provider and clinician workflows? What is the potential for generative AI to assist in improving health outcomes?	The information needed to make medical decisions (eg, medical history, laboratory and imaging results, and unstructured clinical notes) can be scattered across multiple records that exist in myriad formats and locations. Generative AI could be used to compile and organize this information—and put it into a format that is accessible and clinician-friendly—to accelerate and augment critical thinking. In addition, generative AI–enabled ambient documentation could pull information from clinician conversations and generate natural-sounding notes. Technology could also be trained to identify patterns that are too subtle for a human to recognize.
Patients or consumers	What is the consumer or patient perspective on using generative AI for health care? In what ways can generative AI improve the patient experience or patient engagement to manage chronic conditions?	Accurate real-time audio and SMS text messaging could be generated instantly, and in different languages, as frontline workers interact with people for health care and social services. Generative AI also could translate documents, websites, laws, regulations, and policies. Health advisories could make essential information accessible to a diverse population. Generative AI could also play a central role in optimizing and mitigating health and safety risks by generating worksite-specific safety training that replicates real-world settings and critical scenarios.
Frontline or first responders and public health, community social services use of AI	How might generative AI be used to improve patient engagement (from the public health perspective)? In what ways can generative AI be used to streamline emergency response in the field, urgent care, or the hospital emergency department?	Operational inefficiencies or limited capacity in the call center can translate to decreased customer satisfaction. Generative AI could help to create hyperpersonalized experiences with customers and patients. It could also help efficiently support customers and reduce call volume handled by associates. The technology might also assist human staff in generating responses to customer questions, insurance coverage, and other plan details. The customer service experience can have a direct impact on patient perception, even without any change in charged costs or appointment wait times.
Ethical use of generative AI	How can organizations create an ethical framework for generative AI in health care (consider bias and hallucinations)? How can they use an ethical framework and still support team science and innovation? What are the data governance policies and steps needed for quality control and validation of a generative AI model?	Set up an experiment that identifies and examines these issues. Recommendations for how organizations should proceed to build this into governance policies for generative AI and how it should be considered in building capabilities for using generative AI to increase AI literacy in the workforce.

All students enrolled in the course were given the opportunity to participate in the research at the beginning of the semester. Participation was voluntary, and students who chose not to do the research were still able to complete the course.

All data were collected via the Blackboard Learning Management System (Anthology Inc). In Blackboard, students completed a pretest to assess their baseline knowledge of generative AI. The same questions were given as a posttest at the end of the course to assess student learning. The purpose of the pre-post test was to determine any familiarity or knowledge of basic AI concepts. The questions were adapted from LinkedIn Learning courses available to UIC students. Questions were also generated by using ChatGPT with this prompt (Textbox 1):

Textbox 1. **Prompt and sample questions**

Provide 12 questions about generative AI that can be used as a pretest to assess health informatics graduate students' current understanding of generative AI. Make the questions multiple choice, true or false, and make 2 of them short answer. Provide the answer key and any citations that support the questions and that students can go to for more detailed information on the question.

Sample questions were as follows:

1. What is generative AI?

a. A type of AI that focuses on generating new data similar to existing data

b. A method for organizing data

c. A technique for visualizing data

d. A process for analyzing big data

Answer: a

2. Which of the following is a common application of generative AI in health care?

a. Predicting stock market trends

b. Generating synthetic medical images for training purposes

c. Analyzing social media trends

d. Managing electronic health records

Answer: b

3. Generative AI can only be used for creating images and text. True False

Answer: False

4. Describe one way generative AI can be used to improve health care outcomes.

In addition, students completed self-reflections about skills developed and their attitudes toward using generative AI and how they thought it might impact their health informatics work and careers. The reflections were also completed at the beginning and at the end of the semester (Table 2).

**Table 2.** Student reflections.

Reflection	Questions
Reflection 1	As you look to the future, to what degree do you think generative AI^a^ will be useful to your work in health informatics? To what degree do you think it might become part of your work process? What generative AI skills do you think will be most valuable to you in your health informatics career? To what extent do you think generative AI has the potential to enhance your ability to do health informatics work or enhance your productivity? What concerns do you have about using generative AI in your professional work? Are there aspects of generative AI that you anticipate might be problematic? What do you see as some of the drawbacks or challenges of generative AI use?
Reflection 2	Describe how using generative AI for your capstone impacted your satisfaction with the work you produced. Do you think you were more or less satisfied with your results than you would have been without using generative AI? Did you experience disappointment, worry, or anxiety about using generative AI to augment your capstone project? Describe any way in which using generative AI was disappointing or led to worry for you. Did this change over the course of the semester? What impact did using generative AI have on your critical thinking as you worked on your capstone? Did it enhance your critical thinking? Did you experience reduced critical thinking due to overreliance on AI technology? Overall, what was the best thing (the thing you enjoyed the most) about using generative AI for your capstone project? What was the worst thing (thing you enjoyed the least) about using generative AI for your capstone? Please add any additional comments you would like to make about your learning experience and the use of generative AI.

a AI: artificial intelligence.

### Student Assignments and Projects

For their projects, UIC students completed foundational readings. They conducted additional research exploring their chosen topic and developed prompts to use generative AI to assist in their research, brainstorm, and synthesize information. Students then drafted a 1-page description of the project scope and deliverables (final end-products) along with references that support their ideas for approval and feedback. For the rest of the semester, student projects were submitted at defined checkpoints during the semester and faculty provided feedback to guide the projects’ development.

The generative AI projects in the capstone course were individualized, and faculty mentors guided students to do work that would explore their topic and use generative AI specifically for tasks such as brainstorming, research, policy and tool development, and prototype design. For example, for a project on *Ethical Implementation of Generative AI in Primary Care*, the student used a generative AI tool to generate a list of policies that the organization would need to revise or develop to establish organizational governance for using generative AI in primary care. This student then used generative AI to generate a list of roles and job titles that should be considered to participate in a subcommittee for generative AI governance. The student developed a governance structure diagram based on this research. Finally, a clinic-wide training program was developed with generative AI assistance that included basic awareness, role-based training, and ongoing support.

At USF, HS 633 introduces students to generative AI in digital health informatics through a scaffolded blend of technical foundations and applied ethical inquiry. Students are not expected to have prior coding or AI experience; instead, we begin with accessible primers on transformer architectures, natural language processing pipelines, and prompt engineering, grounding each concept in real-world health and ethical use cases. Alongside this, students engage in critical discussions about bias, transparency, and the societal impacts of large language models in clinical and public health contexts. Some of the classroom learning activities include conducting a literature review, interrogating deployment case studies, and prototyping generative AI–informed interventions and workflows.

The capstone assignment asks each student to design a credible generative AI deployment for a real-world clinical or operational problem—one that not only demonstrates feasibility but also foregrounds ethics, equity, and sustainability. Students articulate the problem space, quantify the opportunity, and craft a value-aligned solution that fits into existing health care infrastructures. The major components include market sizing using Total Addressable Market, Serviceable Available Market, and Serviceable Obtainable Market frameworks to assess reach and adoption pathways; ethical charter that explicitly addresses risks around privacy, bias, transparency, and Health Insurance Portability and Accountability Act (HIPAA) compliance—with a mitigation plan for each dimension; evidence-based value proposition detailing projected outcomes, cost savings, and a 5-feature comparison against both legacy workflows and rival technologies; quality measurement strategy, including a 10-item Likert survey aligned to interoperability standards like Fast Healthcare Interoperability Resources, Health Level Seven International, or Systematized Nomenclature of Medicine—Clinical Terms; technical blueprint and product requirements document describing integration touchpoints with electronic medical records, mobile apps, and telehealth systems; scenario analysis exploring best- and worst-case implementation paths, resilience under public health emergencies, and tailored strategies for historically underserved populations; and scenario analysis exploring best- and worst-case implementation paths, resilience under public health emergencies, and tailored strategies for historically underserved populations.

- Technical blueprint and product requirements document describing integration touchpoints with electronic medical records, mobile apps, and telehealth systems
- Scenario analysis exploring best- and worst-case implementation paths, resilience under public health emergencies, and tailored strategies for historically underserved populations

Final deliverables include a written paper, a concise pitch deck, and a 10-minute oral defense. At the core of the assignment is an ethical charter that addresses privacy, bias, transparency, security, and HIPAA compliance, accompanied by a mitigation plan for each identified risk. Deliverables comprise a 15-to-20-page American Psychological Association–formatted concept paper, a 12 to 15 slide pitch deck with an appendix dossier, and a live scholarly defense. Assessment emphasizes the clarity of the argument, the rigor of ethical analysis, and practical feasibility, equipping graduates to articulate and implement responsible generative AI innovations in contemporary health informatics practice. Students are not only conversant in generative AI’s capabilities and limits, but they are equipped to lead conversations at the intersection of innovation, governance, and health equity.

Knowledge was assessed using multiple choice and short answer tests. A pretest was given at the start of the semester, and an identical posttest was completed at the end of the semester. The tests were open book, administered on a learning management system with unlimited attempts. However, students were advised that these tests had no bearing on their grade for the course but were only for research purposes to get a baseline of what students know about generative AI.

An important part of this study was to identify skills that students will need in the workforce. AI skills and attitudes were identified using student reflections on student engagement topics exploring how they would use generative AI in their future career, concerns with using generative AI, and their satisfaction with the end product for their courses [12] At UIC, reflections were given at the beginning and end of the semester.

### USF

The USF participants for this study are 7 out of 8 students who took the *Generative AI Ethics* course in the fall of 2024. A student absent from the initial survey distribution at the beginning of the fall 2024 semester was excluded from this study. The participants are in their second year of the Master’s degree program in digital health informatics. USF students were introduced in class to the UIC+USF study and explained the purpose, inclusion criteria, privacy, and harm risk per the IRB-approved documentation. The 2 components of the survey (pre- or posttest and reflections) were explained to students. Students were told that participation was voluntary and would not affect their grades.

The initial USF pretest was distributed during in-person class time. Students were given ample time, up to 1 hour, to complete their surveys. Surveys identical to those used at UIC were delivered using the Google Survey app. The USF results were deidentified and remained in Google Cloud until the end of the semester. At the end of the semester, a second survey was administered via Google. This survey had 2 sections. The first section was identical to the pretest at the beginning of the semester. The additional second section was for the reflections of students who completed generative AI semester-long projects. USF’s IRB approved the second test before distribution to the study participants.

The second USF survey, like the first, was distributed during in-person class time, their last before students presented their final semester-long *Generative AI Ethics* projects. Participants were given ample time, up to 1 hour in class to answer and reflect on their semester. The USF participants in the second survey were 6 out of 7 students who took the *Generative AI Ethics* course in the fall of 2024. The student absent from the initial study was excluded from the second survey for consistency. Once USF students completed the second survey, deidentified survey results were uploaded to UIC’s secure box system for analysis.

The UIC capstone experience is a culmination of learning throughout the MSHI program. In their capstone, students personalize their project by pursuing an area of interest to them. This has the advantage of incorporating real-world scenarios and teaches critical divergent thinking and active learning. The USF’s *Generative AI Ethics* course equips students with foundational AI knowledge that they can then bring into their work at the intersection of technology and health care. In both programs, assignment instructions included use of generative AI to support the creation of a final product.

### Applying Established Frameworks

Prior to this study, we examined knowledge domains for health informatics education used in previous work by Krive and Isola [11] to create a data science concentration within the MSHI program, and we adapted the following knowledge domains for AI competencies:

1. Essentials of AI
2. Application of AI to health informatics
3. AI transformations to information and knowledge
4. Organizational change and adoption of AI within the health care organization

We developed a preliminary list of 33 competencies (Multimedia Appendix 1) as part of an effort to capture knowledge and skills employers would be looking for in health informatics graduates. The competencies came from multiple sources, including literature, job market analysis, input from industry experts, and our professional experience.

The current study was a first attempt to actually bring generative AI into the curriculum. We used Lo et al [12] as a framework to explore student reactions to using generative AI and also explore the 3 domains of students’ emotional, cognitive, and behavioral engagement and disengagement with regards to ChatGPT use. Lo et al [13] conducted a systematic review that examined indicators of engagement and disengagement in each domain; they found that much remains to be researched about ChatGPT as a learning tool but that current research shows it leads to both beneficial and concerning emotional, cognitive, and behavioral outcomes [12].

The 23 pre-post test questions were foundational knowledge questions about generative AI that would be used in an introductory course assuming no prior knowledge of generative AI. The reflection prompts were based on Lo et al [13], exploring student engagement with generative AI. Using short answer prompts enabled us to gather qualitative data on student reactions to and experiences with using generative AI. Student responses were then examined against the list of AI competencies (Multimedia Appendix 1) for analysis in seeing what changes in knowledge, skills, and attitudes had taken place. These data can inform programs about key domains and concepts to consider when bringing generative AI into their curricula.

### Main Measures

We collected data about developing student competencies in generative AI (knowledge, skills, and professional attitudes). The research questions were as follows:

1. Did students learn (develop knowledge) about generative AI by doing the assignments?

Regarding knowledge, students completed a pre- and posttest survey with 23 items each worth 1 point. Final scores for pretest and posttest were compared to see if students demonstrated higher scores on the posttest.

2. Did students say they developed generative AI skills and professional attitudes by completing the assignments?

Regarding skills and professional attitudes, student reflection data were collected.

Categorization of student responses was completed by 2 faculty members from UIC and 1 from USF. Each student response was evaluated to select the 1 primary element it contained and then was assigned to 1 competency category. Responses that were not related to the subject received no assignment (eg, “thank you” and “happy I took the class”). Due to this variety of the responses we received in terms of the content, depth, and clarity, we evaluated and counted all applicable student reflections across all domains. This method of evaluating all eligible responses (and excluding the most basic ones) from students explains why our numbers presented in the results exceed the number of students in the UIC and USF cohorts. Differences in faculty categorization were examined together in a work session to develop a consensus. To help us come to a consensus, we used some basic rules, that is, reflections related to ethics or accuracy were assigned to the AI transformations to the information and knowledge domain; and for longer student responses with multiple perspectives, raters assigned the knowledge domain that they agreed was the predominant domain. In most instances, such basic rules enabled us to arrive at an easy consensus with a higher degree of reliability, while in a smaller number of instances, we needed a larger discussion around the real meaning behind student reflections. The latter discussion component introduced minor variability to the qualitative outcomes of our work, since some student reflections were comprehensive and could be interpreted in more than one way.

## Results

### Pre-Post Test: Quantitative

Students completed a pretest survey of 23 multiple choice and short answer questions. Students demonstrated an improvement in knowledge from pretest to posttest in both courses, with UIC students improving from 81% to 93% at the end of the capstone course. USF students also improved from 77% to 80% by the end of the *Generative AI Ethics* course.

### Reflections: Qualitative

To inform revisions to the preliminary list of skills, reflections were rated by 3 faculty members individually followed by a group review and consensus-building session using the knowledge domain definitions and preliminary list of skills (Multimedia Appendix 1). Results of the faculty classifications of student responses into the 4 knowledge domains are summarized in Table 3.

**Table 3.** Faculty classifications of emerging generative themes from student reflections.

Knowledge domain	UIC^a^ faculty 1	USF^b^ faculty	UIC faculty 2
Essentials of AI^c^	28	28	27
Application of AI to health informatics	14	14	14
AI transformations to information and knowledge	38	38	38
Organizational change and adoption of AI within the health care organization	23	23	23

a UIC: University of Illinois Chicago.

b USF: University of San Francisco.

c AI: artificial intelligence.

## Discussion

### Generative AI in Health Professions Education and Comparisons With Prior Work

The latest generative AI technologies are forging ahead and aim to change the ways students in health professions are educated [13] and medicine is practiced by emphasizing personalized care delivery based on individual characteristics of each patient derived from a wide variety of sources [14]. These technologies have already entered the medical field, whether educators are ready to address their impacts or not. So, despite faculty concerns over ethics, cheating in classrooms, and adjusting assignments to the current realities of the knowledge sources available to students, educators must ensure that generative AI is incorporated into the classroom. AI-assisted medical offices are already a reality for many health professions, and employers will have an expectation of this knowledge. The dialog about skills to be taught in the classroom, which is typically the combination of technical, medical application, and ethics perspectives [15] has already started via surveys of educators and practitioners [16], and analysis of the existing and upcoming programs and courses in clinical informatics [17].

Academic leadership in several fields of medicine has initiated inquiries into potential curriculum updates to embed AI, whether as a single course dedicated to AI or as modules embedded into existing courses to infuse AI competencies into curriculum [15,18, 19 20]. Research has explored how AI changes exam taking by comparing the performance of AI versus students on structured multiple-choice tests in medical specialties, subspecialties, and even a basic medical informatics course [20, 21]. These are interesting and valid perspectives for inquiry that will inevitably result in a solid basis for embedding generative AI into curricula and understanding how it will change classrooms of the future. However, educators have a more immediate task at hand—how do we prepare our graduates to face the realities of professional practice and employer expectations today, when the job market already requires generative AI skills? A productive dialogue among educators leading to future methods and models of education will not prepare current students for market realities. Therefore, while today’s methodological efforts will result in strong foundations and understanding of the generative AI role in the classroom in the longer-term future, we assessed what could be done now to prepare our students for AI realities of the present day.

### Principal Results

Differences between pre- and post-tests were noted, with higher scores achieved on the posttest. However, due to the open-book nature of the tests, performance increases may reflect the use of resources or research skills. The UIC capstone course is a research course, and students may have done research on the subject prior to and during the initial testing, despite instructions to use their existing knowledge for pretesting. Higher scores on the majority of the tests do indicate a high level of student engagement and interest in the subject, and if nothing else, these tests may have served to create interest in generative AI research among students. Because HS 633 is a novel course at USF that introduced students to generative AI concepts, emphasizing ethical frameworks rather than the technical details covered by the knowledge test, its content aligned less directly with the test domains, resulting in a smaller score gain for USF students.

A variety of perspectives was observed in the responses to the student reflection questions. Student reflection questions were developed to capture a broad range of potential reactions to using generative AI tools and asked about various dimensions of student engagement in behavioral, emotional, and cognitive domains as defined by Lo et al [13]. Students reported both satisfactions and dissatisfactions with using generative AI. Satisfaction can support creativity and innovation, build enthusiasm for the subject, and stimulate emergence in learning. This feature makes it worthwhile for educators to incorporate it into assignments. Responses included statements such as:

Excitement about building something and feeling empowered to build it. In the past, I would have “big” ideas that felt impossible to see through or felt like it was “just a dream.” Now, with Generative AI, it’s like building it can be a reality.

Being able to experiment! I felt like keep on writing more and more prompts and keep reading the answers, that way I invested more time in the project and was able to dive in deeper in my project.

Satisfactions were related to (1) the process of using generative AI, that is, “it helped me get the ball rolling with my project,” and (2) satisfaction with results, that is, writing a paper with a higher degree of confidence. There are several other observations of satisfaction, as follows:

- Generative AI helped to brainstorm and be more creative (“it brought the best side of me”).
- Generative AI allowed me to focus on critical analysis while minimizing time spent on tedious tasks. It helped me to evaluate the analysis, improve the quality (“this is doing it better, not simply faster”).
- Saved time and efficiency (“the point of using Generative AI is NOT just so you can do it faster, but it is so you can do it better-saving time allows you to do it better”). This must be explicitly conveyed to students and be part of the design of the assignments.
- Just to be trying it out (“as something new, it deserves exploration; it is a gift to be in on something exciting and new”).

Dissatisfactions were more prominent early in the use of generative AI, and with experience and time, concerns decreased as students developed ways to cope with and address these concerns. Some examples of dissatisfaction were:

1. Using generative AI will be seen as cheating. Faculty should address this concern with students directly upfront, to clarify their requirements and expectations for using generative AI as a research tool in the learning process.
2. Concern about the impact on critical thinking, how to keep generative AI in the augmenting rather than dominating role. This was not a shared concern mentioned by most students, yet it is a legitimate and important one to bring up.
3. Lose originality of my work, become too reliant on generative AI. This is where students need help from faculty to understand the dividing line between using generative AI for background research versus becoming fully reliant on it to do the work and simply regurgitate and synthesize commonly available knowledge available on the Internet.
4. Inconsistency in generative AI’s outputs, trusting the output. This aspect is good to address at the formative stage for building the initial generative AI skills, such as prompt engineering, or coming up with the right questions, so students feel empowered to use generative AI with confidence and to the best of their technical ability.
5. AI causing harm to patients, which is an ethical concern over the validity of the information provided by generative AI and the safety of patient information made available to generative AI.
6. Decreased anxiety, which is ultimately a positive factor in supporting student learning.

We also observed the following differences and similarities between UIC and USF student cohorts:

1. UIC students were more focused on clinical applications and process or ethics knowledge domains 2 and 3.
2. USF students were more focused on the technical and process or ethics knowledge domains 1 and 3.
3. Domain 3 (process or ethics) is the common domain of interest between students from both institutions.
4. USF students have a distinct technical focus, in addition to concerns over ethics and validity of the outcomes or information.
5. UIC students have a distinct clinical application and process focus, in addition to concerns over ethics and validity of the outcomes or information.

These differences could be attributed to variances in demographics and professional orientations of students, with USF graduates interested in positions in technology and consulting, and UIC students primarily expecting to be employed by health care providers, community, and public health organizations.

Student reflections were open format, allowing them to reflect on topics of their choice, and they frequently commented on more than one knowledge domain in a single response. This made reflections open to interpretation, and the evaluating faculty differed in how they categorized them. Using basic rules and the process of coming to a consensus among the three faculty who evaluated reflections helped us minimize the differences between faculty perceptions and normalize the numbers, leaving few differences between faculty classifications. We pursued this normalization process to be able to report on the top knowledge domains of interest by students together and on behalf of both USF and UIC institutions, as quantified by faculty based on which topics students focused on in their course reflections.

Table 3 shows the distribution of student responses across the domains. The data reveals that students responded more often to the topics of ethics, knowledge generation, organizational efficiency, and technology essentials, making them a high priority or area of interest or concern for our students. These interests consequently fall under domain knowledge 1 (essentials of generative AI) and domain knowledge 3 (ethics and knowledge of generative AI), followed closely by domain knowledge 4 (organizational change and efficiency). These choices by students indicate a high level of concern over proper technology selection and accuracy or appropriateness, compared to clinical applications, with organizational applicability and driving efficiencies as a secondary area of interest. A lower level of concern over application to health informatics is interesting, given that students are engaged in the informatics dialog and pursuing graduate education in health informatics. However, this might be explained by higher concern over proper selection and implementation of these technologies, which must be solved before medical applications are pursued with provider and patient safety in mind.

The competencies taught in accredited health informatics programs were examined recently by El-Gayar et al [22]. This group looked at the gaps in CAHIIM-accredited HI curricula competencies compared to skills/competencies employers are seeking in actual jobs posted on Indeed.com. They found that educational programs tended to focus on foundational competencies, while the job market demands were more focused on practical applications. Forty-six specific competencies were identified as gaps needed to prepare the health informatics workforce.

This analysis raises some questions about how well programs' stated competencies capture exactly what we are teaching in the courses at the assignment level. Competencies for the program may be defined more broadly and do not capture regular updates faculty make to courses and assignments. Programs may already be teaching some of the competencies El-Gayar et al [22] identify as gaps. However, this analysis also points out that areas of expectations between programs and employers may be disconnected. It is a useful starting point to inform further research and discussions about curriculum, with the goal of aligning Commission on Accreditation for Health Informatics and Health Information Management Education accreditation competencies, employers’ needs with regards to AI and other knowledge and skills, and what instructors are actually teaching.

### Limitations

This work was a collaboration between 2 distinctly different institutions of higher education in Illinois and California based on the common interest of swiftly bringing generative AI into the classroom. Our programs differ by student demographics, goals, and mode of education (hybrid vs online attendance). This allowed us to test various methods in 2 different academic environments. The cohorts of students were relatively small, and the courses were offered for one academic term, with plans for expanded offerings informed by these findings. We derive strengths by having two distinctly different academic environments and speed of innovation (summarizing results to quickly share with faculty colleagues around the world) and face limitations with our approach (small cohorts not allowing advanced statistical analysis, lack of identical course offerings, different student cohort characteristics). Demographic differences between our student cohorts introduce challenges to group comparisons and influence outcomes, making comparison methods potentially inaccurate. The knowledge test was foundational and may not equally reflect learning in both courses. Due to differences between the programs, we must be careful in making direct comparisons of outcomes between UIC and USF. However, these differences also represent opportunities to widen the scope of our work and generalize it to more schools and faculty looking to learn from our experience of introducing generative AI in various health informatics learning settings and to a wider audience of students. These factors could be excellent subjects of future scholarly inquiry.

Attrition of two UIC students at posttest indicates that they did not choose to complete the posttest. Even with a reminder message, those 2 students did not complete the posttest. We agree that this could also skew any reported improvement. One USF student was absent during the proctored, in-person posttest session and later requested to take the survey; this student’s request was declined to avoid introducing uncontrolled variables. Both USF’s before and after surveys were completed on students’ laptops under faculty supervision, making it reasonable to assume they did not consult external materials while responding.

These 2 programs share a common goal of embedding generative AI into curriculum by teaching students to use cutting-edge technologies in a safe academic space where experimentation is both acceptable and highly encouraged. Thus, we demonstrate a practical approach for bringing generative AI into the classroom to prepare students to meet employer needs and the realities of future practice of health informatics. Therefore, implementation of generative AI assignments into the curriculum is beneficial to students immediately by building competencies, and it can inform faculty in the further development of the health informatics curriculum.

Our assessment of the students’ first foray into generative AI was achieved through a combination of measurements and the subsequent dialog. Therefore, we report knowledge assessments as data and reflection responses in this discussion. More research would be necessary to study the introduction of additional assignments and measuring outcomes of their work, as generative AI curriculum continues to develop. However, this initial work to introduce practical generative AI curriculum in the graduate health informatics programs arms faculty with a glimpse into the potential successes and challenges of teaching generative AI and provides information on student perspectives which can further inform curriculum updates.

We did not formally calculate an interrater reliability statistic before engaging in dialog about the student reflections. Our faculty dialog and normalization of student reflections analyses to reach near-consensus on outcomes in identifying areas of interest and concern introduced a bias resulting from the ability to see each other’s classifications by knowledge domain. This being an initial attempt to rapidly infuse AI into our curriculum and a first exploration of our qualitative findings, we contemplated this bias and ultimately decided that faculty dialog offered the greatest opportunity to arrive at usable results. We chose this bias as a better form of dialog to enhance usability and generalizability of our academic project and its reported outcomes. Still, we acknowledge that this bias may have influenced our results toward preestablished expectations and produced inaccurate overall ratings of student responses. Future research could include interrater reliability and blind ratings of student reflections to strengthen the rigor of the qualitative findings.

### Implications of This Work on Building the Future Health Informatics Curriculum

Our success in implementing a modular approach to introducing generative AI within a larger health informatics context, and considering other AI implications in health care, has implications for health informatics education. Our work shows that health informatics programs may adopt generative AI competencies into their existing curriculum and then use them as guidelines in designing competency-building learning experiences in generative AI [7]. Existing courses can be modernized by rapidly infusing generative AI (and the overall AI) curriculum into the existing lectures and assignments, thus reducing the administrative overhead associated with a major effort to revamp the curriculum. Limited faculty time can be spent rapidly introducing generative AI to students as a hot-market skill. Major program revisions could also have implications for health informatics accreditation standards. To address employer desires for application skills, a next-level accreditation might ultimately require programs to go further into “doing” on the Miller Level of Competence, perhaps reaching an 80‐20 ratio of Doing to Knowing [22].

We propose rapid targeted curriculum updates as a productive form of delivering high-demand knowledge to students. Moreover, such a targeted approach may be the optimal route for all health professions educators to use when updating curriculum with generative AI content, due to limited resources within those programs which must also adhere to their accredited standards of education.

Taking a modular approach to updating the health informatics curriculum would also enable programs to assess the impacts of the changes from results received from small cohorts of students, because they would be able to introduce updates more rapidly and engage more students. Consequently, sample sizes for curriculum research studies would rise beyond a pilot in this initial study and provide deeper understanding of how generative AI, and AI education overall, impacts student satisfaction and preparedness to enter the job market upon graduation.

### Implications of This Work on the Health Professions Curriculum

While we focused our efforts on the cohorts of health informatics students, our experiences and findings are generalizable to wider student populations preparing for careers in several health professions, particularly nursing, public health, pharmacy, and medicine, but also applicable to other health professions. The entire health care delivery system worldwide will be transformed by generative AI, and all programs are interested in updating their curriculum to include AI and prepare their students to face technological realities of the digital health care workplace. Some programs may decide to create entire courses dedicated to AI, while others will adopt modular approaches by embedding applicable AI topics across multiple courses. We presented 2 distinct ways of embedding generative AI content into the curriculum, so programs looking for a modular approach could learn from USF experience, while those looking for a deeper dive into the generative AI topic may learn from UIC experience of running a dedicated generative AI laboratory. Our 4 knowledge domains and skills-based approach to learning can be adopted by any health professions programs, while introducing clinical use cases and examples which are more unique to the subjects they teach.

### Conclusions

Proliferation of generative AI and digital health education in the health professions curricula is in the early stages and is poised to grow into a large-scale academic debate about implementation, with consensus on the need to embed elements of AI expected from professional societies and graduate education commissions. Therefore, the need to define AI competencies and include them among educational outcomes stretches beyond informatics programs, expanding into the existing health professions [23] as well as emerging specialties such as Master of Digital Health [24,25]. There are ongoing attempts to define a clear set of skills to be taught in such digital health programs, with many of the grassroots efforts originating from experiences of the faculty [23]. By beginning with the AI competencies and designing learning experiences based on them, we innovatively addressed a gap in generative AI education in a health informatics program, tested 2 distinct cohorts of students in both online and hybrid content delivery programs, and shared our experiences and recommendations for rapidly infusing generative AI into the curriculum. These recommendations are generalizable to a wide variety of informatics and health professions education programs, to address the growing need for academia to update programs with the latest technology developments in clinical care and life sciences [24,25].

## Supplementary material

10.2196/76507 Multimedia Appendix 1 Generative artificial intelligence (AI) competencies.

## Abbreviations

AI: artificial intelligence
HIPAA: Health Insurance Portability and Accountability Act
IRB: institutional review board
MSHI: Master of Science in Health Informatics
UIC: University of Illinois Chicago
USF: University of San Francisco
