# Supplementary material for: Incorporating Generative AI Into a Health Informatics Curriculum to Build 21st Century Competencies: Multisite Pre-Post Study
Source: JMIR Med Inform. 2025 Dec 16;13:e76507. doi: 10.2196/76507 (PMC12707436; doi:10.2196/76507)
Supplement: Multimedia Appendix 1 [file medinform-v13-e76507-s001.docx]

## Appendix A: AI Competencies

| ***Essentials of AI*** | ***Application of AI to Health Informatics*** | ***AI Transformations to Information and Knowledge*** | ***Organizational Change and Adoption of AI within the Healthcare Organization*** |
| --- | --- | --- | --- |
| Competencies address the foundational knowledge of AI. Assessments teach students to understand, define and describe concepts and recognize healthcare problems that can benefit from data science solutions. | Competencies address application of knowledge about data, databases, statistics, and programming. Assessments teach students to apply foundational knowledge and develop real-world skills in data science. | Competencies address analytics and interpretation of results. Assessments teach students to design and develop AI products and tools to evaluate and communicate results effectively. | Competencies address the collaboration with stakeholders to make AI a strategic asset for the organization. Assessments teach students to make AI actionable for decision-making to meet strategic goals of an organization. |
| 1. Describe how advances in technology enable AI 2. Locate sources of data relevant to AI 3. Communicate AI-derived insights effectively to technical and non-technical audiences. 4. Awareness of technologies and applications of Natural Language Processing 5. Demonstrate ability to fact-check gen AI results (checking consistency, errors and quality) 6. Understand how to use gen AI to support (not replace) human reasoning 7. Recognize where gen AI can be applied in clinical workflows, administrative processes, and research. 8. Format output of GPT in a presentation to stakeholders 9. Understand laws and regulations (e.g., HIPAA, GDPR) governing the use of AI in healthcare. | 1. Identify when use of AI tools and techniques is appropriate 2. Perform prompt engineering to optimize AI outcomes. 3. Design an experiment to develop a new AI algorithm or product 4. Apply modeling and validation problem-solving processes to provide insight into real-world problems and AI solutions 5. Demonstrate basic diagramming of network and infrastructure for AI systems. 6. Analyze standards and security implications for AI products and data 7. Create custom GPTs to help solve healthcare problems 8. Develop workflows for identifying AI-generated hallucinations or errors. 9. Develop chatbots, and LLM integration into healthcare systems. 10. Apply techniques to identify, address, and document bias in AI-generated results, 11. Generate a synthetic dataset | 1. Critically explore, analyze and interpret AI output 2. Design AI products and solutions using machine learning and Generative AI 3. Develop and evaluate AI policies for health informatics practice. 4. Conduct AI activities aware of and according to policy, privacy, security and ethical considerations 5. Identify potential ethical challenges (e.g., hallucinations, bias, fairness) associated with generative AI use. 6. Understand how generative AI interacts with FHIR, HL7, and other data standards for health systems integration. 7. Adjust workflows to ensure safe and effective use of generative AI results. | 1. Describe how AI facilitates decisions and actions 2. Evaluate socio-technical and ethical dilemmas unique to cognitive computing and artificial intelligence 3. Foster teamwork among clinicians, data scientists, IT professionals, and patients to ensure AI systems deliver optimal outcomes. 4. Cultivate a collaborative mindset to combine AI outputs with human reasoning. 5. Demonstrate how AI can strategically position a health care organization 6. Identify opportunities for development of AI products to support healthcare organization strategies |
